# Microbiome Studies from Saudi Arabia over the Last 10 Years: Achievements, Gaps, and Future Directions

**DOI:** 10.3390/microorganisms9102021

**Published:** 2021-09-24

**Authors:** Khalid J. Alzahrani

**Affiliations:** Department of Clinical Laboratories Sciences, College of Applied Medical Sciences, Taif University, Taif 21944, Saudi Arabia; ak.jamaan@tu.edu.sa

**Keywords:** microbiome, metagenome, health, environment, surveillance, novel bioactive compounds, antimicrobial resistance, food processing, novel strains, space biology, Saudi Arabia

## Abstract

In the past ten years, microbiome studies have shown tremendous potentiality for implementation of understanding microbiome structures and functions of various biomes and application of this knowledge for human betterment. Saudi Arabia is full of geographical, ecological, ethnical, and industrial diversities and scientific capacities. Therefore, there is a great potential in Saudi Arabia to conduct and implement microbiome-based research and applications. However, there is no review available on where Saudi Arabia stands with respect to global microbiome research trends. This review highlights the metagenome-assisted microbiome research from Saudi Arabia compared to the global focuses on microbiome research. Further, it also highlights the gaps and areas that should be focused on by Saudi microbiome researchers and the possible initiatives to be taken by Saudi government and universities. This literature review shows that the global trends of microbiome research cover a broad spectrum of human and animal health conditions and diseases, environmental and antimicrobial resistance surveillance, surveillance of food and food processing, production of novel industrial enzymes and bioactive pharmaceutical products, and space applications. However, Saudi microbiome studies are mostly confined to very few aspects of health (human and animal) and environment/ecology in last ten years, without much application. Therefore, Saudi Arabia should focus more on applied microbiome research through government, academic, and industry initiatives and global cooperation to match the global trends.

## 1. Introduction

In past 15 years, remarkable advancements have been accomplished in the field of metagenomics due to the availability of low-cost sequencing technologies and high-end analytical software. Metagenomic approaches have been applied to understand core microbiota, networks and interactions of microbial communities, host–environment interactions and their effect on spatial and temporal changes of microbiota, prediction of functional phenotypes, and co-evolution of the host microbe, etc. of a microbial ecosystem [1]. Metagenome-based information has been successfully applied for the betterment of plant, animal, and human health; improvement of agricultural productivity; and monitoring of ecosystems and environments, among others [1,2]. Similar to metagenome studies, meta-transcriptome, meta-proteome, and meta-metabolome can also be studied for any microbiota [3]. However, metagenome studies so far have been given the most priority and gene array/panels; 16S rRNA gene amplicon, 18S rRNA gene amplicon, whole metagenome shotgun (WMS), and metagenomic next-generation sequencing (mNGS) are most common molecular technologies used in metagenome studies, followed by the use of several bioinformatic tools to analysis the metagenome data for specific purposes [4,5]. Saudi Arabia is full of distinct climatic regions such as wetlands, deserts, seas, etc. [6,7] and is now facing increased non-communicable disease risk factors in its population due to diversity in demography and socio-economic structure [8,9]. Therefore, microbiome-based research outcomes, specifically in the area of health and the environment, could be important for Saudi Arabia to assess the upcoming health- and environment-associated challenges and to develop proper management strategies.

In this review, first an overview on global trends of metagenome-based microbiome studies is discussed (Table 1), followed by literature search specific to Saudi Arabian metagenome-based microbiome studies (Table 2) to present the microbiome studies that have been so far carried out by Saudi scientists. Finally, the gaps in Saudi studies are highlighted and future directions are discussed.

## 2. Global Trends of Metagenome Studies

Metagenome-based microbiome studies have been applicable to many fields, including human health, agriculture, plant pathology, biotechnology, food science, antimicrobial resistance, environmental monitoring, marine biology, astrobiology, etc., to name a few [1]. Table 1 provides an overview of the global focus on metagenome-based microbiome studies.

### 2.1. Global Metagenome Studies on Human Health

Genomes of the microbes and host are called hologenomes and the host–microbiota interactions are one of the major aspects of metagenome studies towards improvement of human health. Studies on diversity and function of healthy human microbiomes started in 2012 by the Human Microbiome Project Consortium [10]. The gut and oral microbiota can be a signature of ethnicity [11,12]. A change of gut microbiota directly correlates with diet and various diseases [13]. Gut microbiota dysbiosis is associated with several diseases including Parkinson’s disease, Alzheimer’s disease, hypertension, atherosclerosis, obesity, diabetes, non-alcoholic fatty liver disease, inflammatory bowel disease, and colorectal cancer, among others [14]. Similarly, dysbiosis of oral microbiota can be a sign of periodontitis, dental caries, oral cancer, esophageal cancer, pancreatic cancer, cystic fibrosis, cardiovascular disease, rheumatoid arthritis, Alzheimer’s disease, diabetes, etc. [15]. Vaginal microbiome composition indicates normal and various gynecological complications [16,17]. Clinical metagenomics have also been successfully applied for infectious disease diagnosis. Array or panel technologies have been used to diagnose pathogens for gastrointestinal diseases from stool samples [18], encephalitis or meningitis from cerebrospinal fluids [19], and respiratory tract infection from lower respiratory tract samples [20,21]. The mNGS technology has been used to diagnose pneumonia [22], sepsis [23], and encephalitis [24]. Therefore, restoration of normal microbiome is a potential therapy for various diseases associated with microbiome dysbiosis, including cognitive impairments [25,26,27]. Similarly, microbiome engineering has also been suggested to improve domestic animal health [28,29]. There are several examples and Table 1 provides an overview.

### 2.2. Global Metagenome Studies on Environment

#### 2.2.1. Metagenome for Environment and Ecology Surveillance

Metagenome-assisted analysis of environmental microbial communities can be employed as a biosensor for monitoring biodiversity and environmental management [30]. The Earth Microbiome Project (www.earthmicrobiome.org) was initiated during 2010 to construct the microbial and microbiome map of the earth. Similarly, the Tara Oceans consortium is investigating the marine ecosystems, microbiome diversity, and microbiome–environment interactions at the genetic, morphological, and functional level of oceans [31,32]. A lot of metagenomic studies have been conducted so far on the environment and ecology. To mention a few, it was reported that the microbiota differs among human-occupied homes, and this microbiome could be unique for each family [33]. The Tongue River sediment metagenome shows a highly enriched microbial ecosystem, and the microbial community structure and functions change in response to anthropogenic drivers near towns, coal and methane by-products [34]. Processes of anaerobic hydrocarbon degradation have been explained by a bacterial metagenomic study of sub-tidal sediments from polar and sub-polar coasts [35]. *Rhodanobacter* and *Rhodocyclaceae* are sensors for the presence of uranium, and *Oceanospirillales* is a good indicator for oil contamination [36]. Metagenome-based analysis of microbial diversity and ecology of soil, river, lake, seashore, mangrove, ocean water and sediments could indicate the metabolic architecture of the specific microbiome and could also be a marker for water quality, various pollutants, and chemical contaminations [37,38,39,40,41,42] (Table 1). Metagenome of water sediment can also be used for public health risk assessment [43].

#### 2.2.2. Metagenome for Surveillance Antimicrobial Resistance

In today’s scenario, antimicrobial resistance (AMR) is a global health and environmental issue. Metagenome can be a valuable tool to identify and monitor antimicrobial resistance genes (ARGs). Metagenome-based analysis reveals that water treatment plants (urban wastewater and sewage) area hotspot for ARGs [44,45]. Metagenome analysis of mangrove microbiome and glacial lake sediments also show the presence of ARGs [38,46]. A comparative metagenomics analysis shows that gut microbiomes of humans and mammals carry the widest diversity of ARGs compared to the metagenome samples from water, soil, plants, and insects [47,48]. Using the metagenome approach, several ARGs have also been identified in ready-to-eat foods [49], fecal microbiota [50], merine fish [51], and dairy and beef production wastes [52] (Table 1). Apart from the known ARGs, metagenome approaches have also been successfully applied to identify novel kanamycin and ceftazidime resistance genes [53].

### 2.3. Global Metagenome Studies on Other Aspects

#### 2.3.1. Metagenome for Food Monitoring

Pathogenic microbial contamination is one of biggest problems in the food industry with food safety. Whole metagenome sequencing (WMS) has been used to detect *E. coli* that produce Shiga toxins in spinach [54]. Pathogenic *E. coli* and *Klebsiella pneumoniae* strains were identified from Ghanaian fermented milk product *Nunu* samples by Walsh et al. [55]. WMS showed that *Lactobacillus*, *Leuconostoc*, and *Weissella* were the predominant genera in Mexican *Cotija* cheese [56]. Diagnosis of foodborne outbreaks can also be monitored by WMS [57]. *L. monocytogenes* that were responsible for a Listeriosis outbreak were identified from ice cream samples using WMS [58]. Microbiome analysis of a beef processing chain showed the presence of production stage-specific shifts of food pathogens such as: *S. enterica*, *E. coli*, and *C. botulinum* [59] (Table 1). The uncultured food-spoiling bacteria *Thermus thermophilus* that is associated with cheese pinking spoilage was identified using the metagenome approach [60]. Metagenome has also been used to understand the food fermentation process [61] and how the microbiota and specific microbial species of fermented foods improve health [62]. Therefore, metagenomics can be a highly useful tool to assess, monitor, and improve food and food industries.

#### 2.3.2. Metagenome for Industrial Applications

Application of metagenomics has tremendous industrial potentiality. Metagenomics-based functional screening of environmental microbiome is an important trend in industrials, with biotechnologies to identify bacterial strains producing ideal biocatalysts, elusive antimicrobial metabolites, and novel industrial enzymes [63] (Table 1). So far, several industry-grade novel cellulases, proteases, lipases, and bleomycin resistance dioxygenase enzymes have been successfully screened and produced using metagenome approaches. Functional metagenomics have been used to discover novel enzymes for food and pharmaceutical industries. Such novel enzymes include low pH thermo-stable alpha-amylase, thermo-stable esterase, cold-active lipase, alkaline-stable family IV lipase, protease-insensitive feruloyl esterase, and cold-active beta-galactosidase, to name a few. Important metagenome-assisted discoveries of novel bioactive and biosynthetic pathways include pederin, biotin, vibrioferrin, Borregomycin A and B encoded by *bor* pathway, serine protease inhibitor, salt-tolerance genes and acid resistance genes. Similarly, several novel antimicrobials, anti-infectives, and antimicrobial resistance genes have also been identified through metagenome approaches. Some examples include turbomycin A and B, chitinase with chitobiosidase activity, lactonases, LuxR/LuxI genes, bacterial NAHLase, carboxylesterase, kanamycin, and ceftazidime resistance genes [53,64]. Metagenomics have also been used to discover novel endoglucanases for production of second-generation biofuel [65].

#### 2.3.3. Microbiome and Astrobiology

Applications of metagenomics are not only restricted to humans, industries, or the earth, but also beyond the earth’s atmosphere. In the last few years, metagenomics has been applied towards safety and space exploration to understand the taxonomic and functional characteristics of microbiomes in extreme conditions and anoxic sites [66]. Metagenomics study has revealed how the minimal genomes and genome plasticity of *Pseudomonas* can thrive under severe nutrient stress conditions [67]. *Corynebacterium ihumii* GD7 was identified by WMS as the dominant species at the International Space Station (ISS) [68]. Spacecraft assembly cleanrooms and ISS microbiomes also showed several pathogenic bacteria, including *Acinetobacter baumannii*, *Haemophilus influenzae*, *Klebsiella pneumoniae*, *Salmonella enterica*, *Shigella sonnei*, *Staphylococcus aureus*, *Yersinia frederiksenii*, and *Aspergillus lentulus*, with several ARGs and cobalt-zinc-cadmium resistance genes [69,70]. Simulated long space flight travel shows increased dominance of *Bacteroides* and *Prevotella* in the astronaut’s gut microbiome [71]. It was also revealed through metagenome analysis that transmission of microbes to ISS or an astronaut’s microbiome happened, and vice versa [72,73]. Metagenomic analysis has similarly been used to understand how and which bacterial community members of the kombucha (a drink) mutualistic community (KMC) survive under a Mars-like environment at ISS [74]. Researchers also have shown how the most dominant species, *K. oboediens*, of space-exposed KMC retains its robustness in cellulose production through its intact cellulose-producing *bcs* operon [75]. Metagenome-based approaches have also been used to isolate and characterize the novel strain *Kalamiella piersonii* gen. nov., sp. nov available only at ISS [76] (Table 1). Therefore, a lot of metagenome applications are possible towards understanding the space environment and exploration.

## 3. Literature Search Criteria and Article Selection to Review Metagenome-Assisted Microbiome Studies from Saudi Arabia

To retrieve literature on Saudi Arabia’s microbiome-related publications, the PubMed literature database (www.pubmed.ncbi.nlm.nih.gov) was searched with the advanced search option using keywords: “Microbiome” (Title/Abstract) OR “microbiota” (Title/Abstract) OR “metagenome” (Title/Abstract) OR “16SrRNA” (Title/Abstract) OR “amplicon” (Title/Abstract) AND “Saudi Arabia” (Title/Abstract). Articles written in English and published from June 2011 to July 2021 were considered. Each retrieved article was manually scanned, and the technology-related articles were excluded, while microbiome studies as per global trends were considered for this review. Following this search criteria, 121 articles were collected, out of which 38 were found to be relevant for this review. When we used the same keywords without “Saudi Arabia” (Title/Abstract), 75,391 papers were found. When the selected articles were classified as per the global trends of microbiome studies, a mismatch was observed. Unlike the global trends, the Saudi Arabian studies were aligned to only two major global trends: human and animal health (13 articles) and the environment (11 articles). However, three articles on camel parasites and one article on date palm pests were found. Additionally, 10 articles were found to describe novel isolates. However, for most of these studies the metagenome approach was not used. Table 2 represents the metagenome-based microbiome studies by Saudi researchers in the last 10 years.

## 4. Saudi Arabian Microbiome Studies on Human Health

Saudi Arabian metagenome studies are limited to obesity, diabetes, autism, infections, and pregnancy (Table 2). Yasir et al. [77] reported significant abundance of *Firmicutes* in fecal microbiota of obese Saudis as compared to their normal weight controls. The gut microbiome analysis by Angelakis et al. showed less species richness and biodiversity in Saudi obese subjects as compared to Amazonians and Polynesians. However, *Lactobacillus* sp. abundance is more prevalent in Saudis than the Polynesians [78]. Two new strains of *Bacillus jeddahensis* sp. nov. (JCE(T) and *Oceanobacillus jeddahense* sp. nov. (S5T) were isolated from a stool specimen of young obese patients by Bittar et al. [79] and Khelaifia et al. [80], respectively. Recently, Kieu et al. reported the presence of new bacterial species *Clostridium culturomicium* strain CL-6^T^ and *Clostridium jeddahitimonense* strain CL-2^T^ in the gut microbiota of an obese man from Saudi Arabia [81]. Using sub-gingival samples and 16S rRNA-based analysis, Al-Obaida et al. reported the presence of *Aggregatibacter actinomycetemcomitans* (43%) and *Capnocytophaga ochracea* (100%) in diabetic patients from Saudi Arabia [82]. Abdulhaq et al. studied the tongue microbiota of children with autism spectrum disorder (ASD) in Jazan city and noted that there was no significant difference in the microbiota compared to that of healthy children [83]. Analysis of bacterial metagenome from blood samples of Saudi Arabian blood cancer patients with bloodstream infections showed the predominance of Gram-negative bacteria (82%) in which *E. coli* and *K. pneumonia* were higher [84]. Based on amplicon and metagenome sequencing, various bacteria associated with pneumonia, namely *Acinetobacter baumannii*, *Pseudomonas aeruginosa* and *Streptococcus pneumonia*, and several ARGs were reported to be present in the oropharyngeal swabs, tracheal aspirates, and throat swabs samples of Saudi patients infected with Middle East respiratory syndrome CoV (MERS-CoV) [85]. 16S amplicon sequencing and the culturomics-based gut microbiota of pregnant and non- pregnant Saudi females were studied by Khan et al. [86]. Both the groups showed the presence of *Firmicutes*, *Bacteroidetes*, *Proteobacteria*, and *Actinobacteria*. However, a sharp decline of *Bacteroidetes* was noticed during the first trimester of pregnant women. On the other hand, relative abundance of butyrate-producing bacteria (e.g., *Faecalibacterium* spp. and *Eubacterium* spp.) were found in pregnant women, whereas *Prevotella copri* was significantly higher in non-pregnant females. The researchers also noted the presence of antimicrobial resistant genes (ARGs) in pregnant women that could be unfavorable for both mother and fetal health [86] (Table 2). Al-Moaleem et al. showed that the oral microbiome of khat- and non-khat-chewing Saudi people from Jazan region predomantly had *Lactobacillus* and *Veillonella* spp. in both the groups. *Lactobacillus* was found to be higher in khat chewers, which was associated with prosthetic materials [87]. Badger-Emeka et al. reported that different doses of vitamin D can influence the body weight and gut microbial colonies in mice. The authors found decreased gut *Salmonella/Shigella* and *E. coli* colonies under low- and normal-dose vitamin D. However, *P. aeruginosa* was significantly decreased under high vitamin D doses [88].

## 5. Saudi Arabian Microbiome Studies on Animal Health

Camel is an important animal in Arabian deserts, and ticks (a blood feeder that can transmit a wide range of microbes and pathogens) are one of the most important parasites of camel. Using 16S rRNA amplicon analysis of ticks from the camels of Hofuf city, Elbir et al. reported 17 microbial species under 114 genera. The camel ticks of this region predominantly showed abundance of phylum such as *Proteobacteria* (98%), *Firmicutes* (1.38%), *Actinobacteria* (0.36%), and *Bacteroidetes* (0.17%). The researchers also identified the bacterial pathogen *H. pylori* in their tick samples [89]. Elbir et al. in another study also reported species diversity among *Francisella*-like endosymbionts (FLEs) and non-specialized circulation of FLEs among 151 *H. dromedarii* ticks that were collected from 33 camels from 13 different locations in Saudi Arabia [90]. Alreshidi et al. carried out a similar study involving 200 ticks obtained from healthy camels in the Al Khotha and Al Gayed regions of Hail province. Their massive 16S rDNA sequencing-based metagenome analysis revealed the presence of several distinct microbial communities from two locations. *Proteobacteria* (61.3%) and *Firmicutes* (31.2%) mostly dominated the ticks from Al Khotha region and *Proteobacteria* (81.2%) and *Firmicutes* (9.2%) were predominant in ticks from the Al Gayed [91]. Such data and findings may have great veterinary and medical importance. Except for these studies, no other metagenome-based microbiome studies by Saudi Arabian authors were found on human or animal health (Table 2).

## 6. Saudi Arabian Microbiome Studies on the Environment

Almost all environmental microbiome studies by Saudi scientists are to report microbial diversity in various ecological samples of the country (Table 2). Alzubaidy et al. first reported the microbial diversity of rhizosphere microbiome (of *Avicennia marina*) from the Red Sea. The authors observed a predominance of *Proteobacteria*, *Bacteroidetes*, and *Firmicutes* in their study [92]. Al-Quwaie et al. reported that the soil rhizosphere microbiota varies depend on the desert wild plants, such as *Calotropis* procera and *Senna alexandrina*. *Streptococcus sobrinus*, *Veillonella parvula*, and *Sphingomonas* genus are enriched in the rhizosphere of *S. alexandrina*. High abundance of *Pseudomonas stutzeri* and *Virgibacillus koreensis* in the soil rhizosphere of desert wild plants *C. procera* provides saline resistance. *Marinobacter*, *Porticoccus,* and *Alcanivorax* genera that are present only in the rhizosphere of *C. procera* protect the plants from pathogen infections [93]. Using 16S RNA sequencing, Yasir et al. analyzed the soil bacterial community along the south-western highlands, which is susceptible to environmental changes. They identified 33 different phyla, among them *Proteobacteria*, *Actinobacteria,* and *Acidobacteria* were most dominant [94]. Moussa et al. analyzed soil samples from four different locations in the Mecca region and reported 460 fungal species that belong to 133 genera, 58 families, 33 orders, 13 classes, and 4 phyla [103]. In another study, Saudi Arabian dust storms showed relatively low abundance of *Actinobacteria* and high abundance of *Proteobacteria* when compared with other dust storms in other countries [95]. However, these two studies did not use the metagenomic approach. Yasir et al. studied the bacterial diversity in sediment samples from six hot springs of Saudi Arabia and reported that the most abundant species were *Bacillus* and *Brevibacillus* [96]. The same research group analyzed microbial communities in mat samples from two hot springs from Al Aridhah and found that the *Chloroflexus* was the most dominant taxa among the diverse group of bacteria identified [97] (Table 2). In both the studies, the authors used 16S RNA sequencing. Li et al. analyzed the microbial communities in managed aquifer recharge (MAR) systems obtained from Taif River (Taif, Saudi Arabia) and South Platte River (Colorado), in which heterotropic *Proteobacteria* were found to be dominant. The authors inferred that the addition of labile-dissolved organic carbon could influence the composition and/or metabolism of these microbial communities [98]. Several genera related to opportunistic pathogens (e.g., *Acinetobacter*, *Aeromonas*, *Arcobacter*, *Legionella*, *Mycobacterium*, *Neisseria*, *Pseudomonas,* and *Streptococcus*) were reported in chlorinated effluent of local wastewater collected from the Thuwal area of Saudi Arabia [99]. Mineralization of crude oil requires organic transformation by bacteria. Albokari et al. reported microbial communities of crude oil and oil sludge samples obtained from Saudi ARAMCO Oil Company [101]. The authors noted prevalence of *Alphaproteobacteria*, *Betaproteobacteria*, *Gammaproteobacteria*, *Clostridia*, *Spingobacteria,* and *Flavobacteria* in sludge samples and *Bacillus*, *Clostridia,* and *Gammaproteobacteria* in crude oil. They also pointed out that *Bacilli* is the most dominant taxa in crude oil and *Flavobacteria* is the most dominant taxa in oil sludge samples [101] (Table 2). Bibi et al. investigated three marine sponges belonging to the species of *Pione vastifica*, *Siphonochalina siphonella,* and *Suberea mollis* collected from the Red Sea in Jeddah. They identified large diverse communities in *S. mollis* with 105 OTUs belonging to the phylum *Proteobacteria* and concluded that the abundance of *Proteobacteria* in sponges may have ecological significance and may be used for environmental monitoring [100] (Table 2).

## 7. Saudi Arabian Microbiome Studies on Other Aspects

### 7.1. Studies on Plant Pests

Date palm is an important economic fruit crop in Saudi Arabia. Date palm root borer *Oryctes agamemnon* causes significant loss of crop productivity [104]. El-Sayed and Ibrahim studied the endosymbiotic bacterial communities of *O. agamemnon* larval mid-gut metagenome and reported the presence of 11 major operational taxonomic units (OTUs), such as *Photobacterium*, *Vibrio*, *Allomonas*, *Shewanella*, *Cellulomonas*, and *Citrobacter*. The endosymbiotic bacterial community found predominantly consisted of *Vibrionaceae*, uncultured bacteria, *Enterobacteriaceae*, *Shewanellaceae*, and *Cellulomonadaceae*. The authors concluded that the presence of these bacteria may play a role in digestion and other developmental functions of *O. agamemnon* larvae. However, the authors did not conclude the role of the identified bacteria in host and pest interactions [102].

### 7.2. Bacterial Novel Strains Isolated from Various Parts of Saudi Arabia

Due to the presence of both sea and desert, the climate of Saudi Arabia can be a source of novel microbes of various importance. Sefrji et al. isolated novel *Mangrovivirga cuniculi* gen. nov., sp of the *Mangrovivirgaceae* family from a bioturbated mangrove sediment on the Saudi Arabian Red Sea coast [105]. The same research group also reported another novel bacterium *Kaustia mangrovi* gen. nov., sp. nov of the *Parvibaculaceae* family from the Red Sea mangrove sediments [106]. Rotting et al. isolated a novel extremophile bacterium species *Streptomyces jeddahensis* sp. nov. that can tolerate 50 °C from the desert soil near Jeddah [107]. Similarly, novel halotolerant bacteria *Siccirubricoccus deserti* gen. nov., sp. nov. [108], *Sphingomonas jeddahensis* sp. nov. [109], *Microbacterium album* sp. nov. and *Microbacterium deserti* sp. nov. [110], *Georgenia alba* sp. nov [111]. *Georgenia deserti* sp. nov [112] were also isolated from various desert samples in Saudi Arabia. Gamma- and UV-radiation-resistant novel *Deinococcus saudiensis* sp. Nov was also isolated from the desert soil of Yanbual Bahr [113]. The genus *Streptomyces,* showing anti-blood cancer activity, was identified from the actinobacterial isolates that were collected from the Al-Jouf desert of Saudi Arabia [114]. However, none of these novel strains were isolated using a microbiome or metagenome approach, and in most cases the utility of these bacteria is unknown.

## 8. Conclusions and Future Direction

The global trend of most microbiome studies has focused on understanding the interactions, functional characterization, and implementation of a microbiome towards improvement of human and animal health, the environment and ecology surveillance, and surveillance of antimicrobial resistance and food chain ecology. Additionally, microbiomes and metagenomics also have important industrial applications and astrobiology. Metagenome-based microbiomes have been successfully implemented for diagnosis and to understand disease mechanisms and treatment strategies (microbiome restoration) for various diseases including several cancers and infectious, metabolic, cardio-vascular, neurological, inflammatory, and gynecological diseases. The second global trend of microbiome studies mainly focused on understanding microbial biodiversity of various environmental conditions, such as air, soil, rivers, lakes, seashore, mangroves, ocean water, etc. The environmental microbiome studies also help in developing biosensors for water quality, pollution, and ecological surveillance. Similarly, environmental microbiome analysis through metagenomics can be an important tool to monitor the global crisis of antimicrobial drug resistance. Metagenomics is also an inevitable tool to monitor food processing setup environments and food quality. The technology has prime importance for diagnosing foodborne pathogens and diseases. Microbiomes and metagenome are successfully used to identify novel biocatalysts, antimicrobial metabolites, and enzymes for industrial production and various industrial uses. Even the microbiome analysis using metagenome is a key tool now for astrobiolocial experiments and space exploration.

As compared to the global trends of microbiome studies discussed above, the initiatives from Saudia Arabia on microbiome-based research are lagging behind. While there are more than 75,391 articles published on microbiomes and metagenomics by the global community in last 10 years, Saudi Arabian authors have published only 121 articles and out of which only 27 articles have used microbiome analysis using the metagenome approach. While the global focus of microbiome studies is on (i) human and animal health, (ii) environment and ecology surveillance, (iii) surveillance antimicrobial resistance, (iv) food and food process monitoring, (v) industrial applications, and (vi) space biology/astrobiology, most of the Saudi Arabian studies are restricted to (i) human and animal health (13 articles) and (ii) environmental microbiomes (11 articles). Additionally, while metagenomics have been used in several cancers and infectious, metabolic, cardio-vascular, neurological, inflammatory, and gynecological diseases globally, the Saudi studies are restricted to only two metabolic diseases (obesity and diabetes), two infections (bloodstream infections and MERS-CoV), and one gynecological condition (pregnancy). The environmental microbiome studies are also minimal in Saudi Arabia (only 11 studies), and are only to understand microbial diversity; however, the oil microbiome study is very unique. Other unique microbiome studies from Saudi Arabia include tick metagenomics (parasites of camels) and metagenomics of date palm root borer *O. agamemnon*. Although Saudi scientists have isolated several novel bacterial strains from various deserts in the country, they probably have not used a microbiome and metagenomics approach.

Therefore, to match the global trends, Saudi researchers need to focus on the untouched areas, such as application of metagenomics in various predominant non-communicable diseases. Dysbiosis of the gut microbiota is associated with several gastrointestinal (ulcerative colitis, Crohn’s disease, irritable bowel syndrome) and extra-gastrointestinal diseases (diabetes, obesity, autoimmune disorders, Parkinson’s disease, autism, multiple sclerosis, infections with multidrug-resistant bacteria, multiple organ failure, etc.) and fecal microbiota transplantation (FMT) is one potential therapy for these conditions [115,116] (Table 1). However, no research has been conducted so far in Saudi Arabia on FMT and the clinical microbiome researchers should focus on this aspect. Similarly, other important areas such as antimicrobial drug resistance, food microbiomes (for example, isolating novel probiotic bacteria from the local food resources in Saudi Arabia), industrial novel product development, and space biology also need to be explored. Importantly for microbiome research, international initiatives and cooperation between countries [117], such as the Earth Microbiome Project (www.earthmicrobiome.org) and Tara Oceans consortium [31,32], as well as university initiatives such as the USF microbiome initiative, Vanderbilt microbiome initiative, etc. are required. As per the available literature, Saudi Arabia has so far not taken any international initiative at the government, university, or non-government level to explore microbiome research. Therefore, to be in the global race of microbiome R&D, the government should make a task force to identify the opportunities in microbiome research in Saudi Arabia. Then, by individual initiative (for instance, the Saudi Microbiome Project) or collaboration with suitable partner countries, allocate funds to the universities/research institutes to conduct the research. Alternatively, the Saudi universities and industries could also come forward with their own ideas and take specific microbiome initiatives, like the initiatives taken by universities from the USA and other countries.

## Figures and Tables

**Table 1:** An overview of the global focus on metagenome-based microbiome studies.

Broad Areas of Global Interest	Sample	Outcome of the Metagenome Study	Ref.
Metagenome studies on human health	Disease diagnosis and management		
	Gut microbiota	Microbiota composition indicates Parkinson’s disease, Alzheimer’s disease, hypertension, cognitive impairments, atherosclerosis, obesity, diabetes, non-alcoholic fatty liver disease, inflammatory bowel disease, colorectal cancer	[14,25,26,27]
	Oral microbiota	Microbiota composition indicates Periodontitis, dental caries, oral cancer, esophageal cancer, pancreatic cancer, cystic fibrosis, cardiovascular disease, rheumatoid arthritis, Alzheimer’s disease, diabetes	[15]
	Vaginal microbiome	Microbiota composition indicates female reproductive health	[16,17]
	Cerebrospinal fluids	Microbiota composition indicates encephalitis, meningitis	[19]
	Respiratory tract	Microbiota composition indicates respiratory tract infection	[20,21]
Metagenome studies on environment	Antimicrobial resistance		
	Water treatment plants	Abundance and diversity of antimicrobial resistance genes found	[44,45]
	Mangrove and glacial lakes sediments	Abundance and diversity of antimicrobial resistance genes found	[38,46]
	Ready-to-eat foods	Antimicrobial resistance genes detected	[49]
	Fecal microbiota	Antimicrobial resistance genes detected	[50]
	Beef production wastes	Antimicrobial resistance genes detected	[52]
	Environmental monitoring		
	Human-occupied home	Microbiome uniquely differs for each family	[33]
	Tongue River sediment	Microbial community indicates presence of town waste and methane by-products	[34]
	Soil microbiome	Presence of *Rhodanobacter* and *Rhodocyclaceae* indicates presence of uranium	[36]
	Soil, river, lake, seashore, mangrove, ocean water, and sediments	Microbial community indicates water quality, various pollutants, and chemical contaminations in respective biomes	[37,38,39,40,41,42]
Metagenome studies on other aspects	Food monitoring		
	Mexican *Cotija* cheese	Predominant genera are: *Lactobacillus*, *Leuconostoc,* and *Weissella*	[56]
	Ice cream samples	Identification of *L. monocytogenes, the causal organism for* Listeriosis outbreak	[58]
	Beef processing waste	Presence of *S. enterica*, *E. coli*, and *C. botulinum*	[59]
	Fermentation samples	Microbial community indicates stage of fermentation and the microbes that are beneficial to health	[61,62]
	Industrial applications		
	Environmental samples	Identification of bacterial strains producing novel biocatalysts, antimicrobial metabolites, and industrial enzymes	[53,63,64,65]
	Astrobiology		
	International Space Station (ISS)	*Corynebacterium ihumii* GD7 is the dominant species in ISS	[68]
	International Space Station (ISS)	Identification of novel strain *Kalamiella piersonii* gen. nov., sp. nov in ISS	[76]
	Spacecraft assembly cleanroom samples	Identification of several pathogenic bacteria, antimicrobial resistance genes, and metal resistance genes	[69,70]
	Kombucha mutualistic community	Enable the understanding of how and which bacterial community members survive under a Mars-like environment	[74,75]

**Table 2:** Metagenome-based microbiome studies from Saudi Arabia in the last 10 years.

Broad Areas of Global Interest	Specific Area of Study	Sample	Outcome of the Metagenome Study	Ref.
Metagenome studies on human health	Obesity	Fecal microbiota	Abundance of *Firmicutes* in obese cases	[77]
		Gut microbiota	Abundance of *Lactobacillus* sp. in obese samples	[78]
		Fecal microbiota	Isolation of *Bacillus jeddahensis* sp. nov. (JCE(T)) and *Oceanobacillus jeddahense* sp. nov. (S5T) from obese samples	[79,80]
	Pregnancy	Gut microbiota	Abundance of *Faecalibacterium* spp. and *Eubacterium* spp. in pregnant women	[86]
	Diabetes	Sub-gingival samples	*Aggregatibacter actinomycetemcomitans* and *Capnocytophaga ochracea* are predominant in diabetic samples	[82]
	Autism Spectrum Disorder (ASD)	Tongue microbiota	Abundance of *Actinomyces odontolyticus* and *Actinomyces lingnae* are increased and *Campylobacter concisus* and *Streptococcus vestibularis* are decreased in ASD	[83]
	Bloodstream infections	Blood cancer patient’s blood sample	Abundance of *E. coli* and *K. pneumonia* in the samples	[84]
	MERS-CoV	Oropharyngeal and throat swabs	Dominance of *Acinetobacter baumannii*, *Pseudomonas aeruginosa*, *Streptococcus pneumonia*, and several ARGs	[85]
Metagenome studies on animal health	Vitamin D deficiency	Mice gut microbiota	Decline of *P. aeruginosa abandance* under high vitamin D dose	[88]
	Microbial diversity	Camel parasite ticks	Abundance of *Proteobacteria*	[89,91]
Metagenome studies on environment	Microbial diversity	Rhizosphere microbiome, Red Sea	Predominance of *Proteobacteria*, *Bacteroidetes*, and *Firmicutes*	[92]
		Rhizosphere microbiota desert	Predominance of *Pseudomonas stutzeri* and *Virgibacillus koreensis* provide saline resistance in desert plant	[93]
		Rhizosphere microbiota desert	*Marinobacter*, *Porticoccus,* and *Alcanivorax* genera provide pathogen resistance in desert plant	[93]
		South-western highlands	Predominance of *Proteobacteria*, *Actinobacteria,* and *Acidobacteria*	[94]
		Dust storm	Predominance of *Proteobacteria* and decline of *Actinobacteria*	[95]
		Hot spring sediments	Dominance of *Bacillus*, *Chloroflexus*, and *Brevibacillus*	[96,97]
		Taif River water	Dominance of *Proteobacteria*	[98]
		local waste water treatment plant	Dominance of several opportunistic pathogens	[99]
		Red sea marine sponge	Dominance of *Proteobacteria* in sponges may be a biosensor for environmental monitoring	[100]
		Oil samples	Prevalence of *Bacilli* in crude oil and *Flavobacteria* in oil sludge	[101]
		*O. agamemnon* larval mid-gut	Presence of *Enterobacteriaceae*, *Shewanellaceae*, and *Cellulomonadaceae*	[102]

## Data Availability

Not applicable.
